# Revisiting level II sleep studies in the era of COVID-19: a theoretical economic decision model in patients with suspected obstructive sleep apnea

**DOI:** 10.1186/s41606-021-00063-5

**Published:** 2021-07-15

**Authors:** Najib T. Ayas, Rachel Jen, Brett Baumann

**Affiliations:** 1 Sleep Disorders Program, University of British Columbia, Vancouver, Canada; 2 Department of Medicine, University of British Columbia, 7th Floor, Diamond Centre, 2775 Laurel Street, Vancouver, BC V5Z 1M9 Canada

**Keywords:** Obstructive sleep apnea, Diagnosis, Decision model

## Abstract

**Background:**

The recent pandemic has made it more challenging to assess patients with suspected obstructive sleep apnea (OSA) with in laboratory polysomnography (PSG) due to concerns of patient and staff safety. The purpose of this study was to assess how Level II sleep studies (LII, full PSG in the home) might be utilized in diagnostic algorithms of suspected OSA using a theoretical decision model.

**Methods:**

We examined four diagnostic algorithms for suspected OSA: an initial PSG approach, an initial LII approach, an initial Level III approach (LIII, limited channel home sleep study) followed by PSG if needed, and an initial LIII approach followed by LII if needed. Costs per patient assessed was calculated as a function of pretest OSA probability and a variety of other variables (e.g. costs of tests, failure rate of LIII/LII, sensitivity/specificity of LIII). The situation in British Columbia was used as a case study.

**Results:**

The variation in cost per test was calculated for each algorithm as a function of the above variables. For British Columbia, initial LII was the least costly across a broad range of pretest OSA probabilities (< 0.80) while initial LIII followed by LII as needed was least costly at very high pretest probability (> 0.8). In patients with a pretest OSA probability of 0.5, costs per patient for initial PSG, initial LII, initial LIII followed by PSG, and initial LIII followed by LII were: $588, $417, $607, and $481 respectively.

**Conclusions:**

Using a theoretical decision model, we developed a preliminary cost framework to assess the potential role of LII studies in OSA assessment. Across a broad range of patient pretest probabilities, initial LII studies may provide substantial cost advantages. LII studies might be especially useful during pandemics as they combine the extensive physiologic information characteristic of PSG with the ability to avoid in-laboratory stays. More empiric studies need to be done to test these different algorithms.

**Supplementary Information:**

The online version contains supplementary material available at 10.1186/s41606-021-00063-5.

## Introduction

Obstructive sleep apnea (OSA) is a common respiratory disease characterized by recurrent upper airway collapse during sleep leading to nocturnal hypoxemia and sleep fragmentation (Laratta et al. 2017). OSA is estimated to affect over 900 million adults globally with over 400 million with moderate to severe disease (Benjafield et al. 2019). Untreated OSA is associated with many adverse consequences including motor vehicle crashes, work related injuries, stroke, and heart attacks (Al Lawati et al. 2009; Hirsch Allen et al. 2020). Diagnosing OSA is important as therapy with continuous positive airway pressure (CPAP) has positive impacts on sleepiness, quality of life, blood pressure, and risk of motor vehicle crashes (George 2001; Patel et al. 2003; Alajmi et al. 2007). It is also a highly cost-effective use of healthcare resources (AlGhanim et al. 2008).

Attended full night sleep studies (polysomnography (PSG)) involve collection of multiple physiologic signals (i.e., eye movements, electroencephalogram, oxygen saturation, airflow, respiratory movements) and is the gold standard for OSA diagnosis. However, costs and access to PSG remain barriers; in some areas, wait times for PSG can exceed months to years (Flemons et al. 2004).

As a consequence, limited channel home sleep studies (Level III) have become more popular. These involve collection of fewer physiologic signals at home (e.g., don’t usually collect electroencephalogram information). Sensitivity and specificity of Level III studies are high, ranging from 80 to 100% (Ross et al. 2000). These studies are particularly useful in confirming OSA in symptomatic moderate to high probability patients (Mulgrew et al. 2007a). Compared to PSG, advantages include reduced costs and increased access; the major disadvantage is the limited amount of physiologic information available. Therefore, their usefulness in more complex patients (e.g. pre-existing cardiovascular and pulmonary disease) is questionable, and they typically cannot detect non-OSA diagnoses accurately (e.g. periodic limb movements, central sleep apnea) (Fleetham et al. 2011). In symptomatic patients with a moderate to high probability of OSA, a negative Level III study should generally be followed by PSG so that OSA diagnoses are not missed (i.e., to exclude false negative tests).

Unattended Level II studies involve recording essentially the same physiologic signals as PSG but in a patient’s home. They have generally been used as research tools (Thomas et al. 2014) and have not been widely used clinically. Costs of unattended Level II studies would likely be between those of Level III and PSG. Due to their unattended nature, one disadvantage compared to PSG is an increased rate of technical failure requiring repeat testing.

The COVID-19 pandemic has radically changed how medical care is delivered across disciplines. This includes increased use of virtual formats to minimize direct interaction with patients to reduce risks to patients and staff. Specific to OSA, many patients may be reluctant to come to the sleep laboratory for their PSG and staff hesitant to attend to these patients because of COVID-19 fears (Ayas et al. 2020). Shifting away from PSG towards more comprehensive home diagnosis of sleep apnea with Level II studies where patients/care givers can set these up at home may help solve some of these issues. We believe that this would be an ideal time to consider how Level II studies should be utilized in OSA diagnostic algorithms.

The purpose of this study was to determine how Level II studies might be used in OSA diagnosis algorithms. The focus was on symptomatic patients where studies are used to rule in OSA so that CPAP therapy can be initiated in positive patients. To do so, we used a theoretical decision analysis model similar to what we have used in a previous study that focused on the role Level III studies (Ayas et al. 2010).

## Methods

For the base case, we considered a symptomatic patient referred for suspected OSA with a pretest probability of OSA of P. We then examined four different diagnostic algorithms to compare the cost per patient evaluated and how these might be affected by changes in baseline variables.

### Initial PSG algorithm: (Fig. 1)

Using PSG as the initial diagnostic test was the first clinical algorithm used. For this algorithm we made the following assumptions:

- PSG was the gold standard for OSA diagnosis (100% sensitive and specific).
- PSG had a technical failure rate of 0.
- Patients diagnosed with OSA were started on CPAP.
- CPAP pressure was identified by a trial of auto-PAP.
- A certain proportion (A) of OSA patients did not improve on CPAP.
- In patients who did not improve with CPAP, a proportion (B) required another PSG (i.e. CPAP titration).
- The cost of PSG was $C.
- The cost of a CPAP trial was $D.

**Fig. 1:**
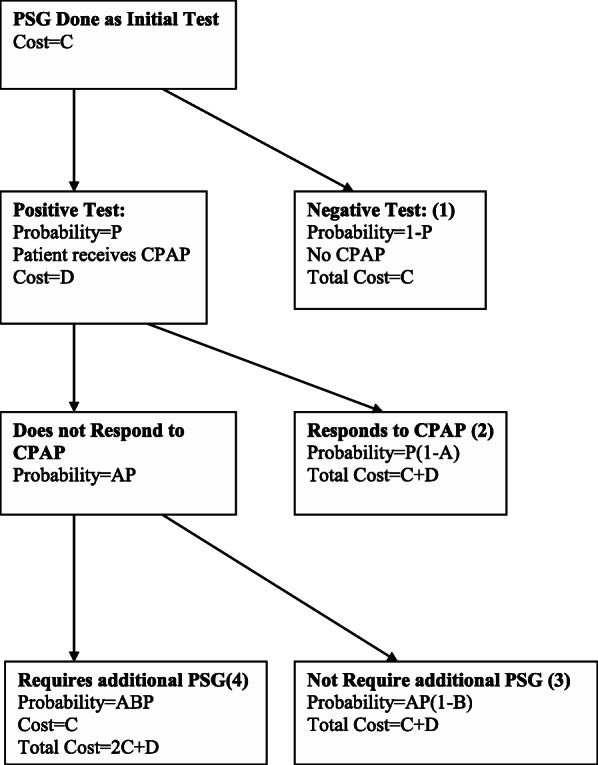
Initial PSG Algorithm. Flow of patients through health states if PSG was used as the initial test

### Initial LII algorithm: (Fig. 2)

For this clinical algorithm, a Level II study was the initial test. We also made the following additional assumptions:

i) There was a failure rate of proportion (J) with a LII study (technical failures, inability for patient to use the device, etc)
j) After failure of an LII study, patients had PSG
k) LII was as sensitive and specific for OSA as PSG
l) The cost of the LII study was $K.

**Fig. 2:**
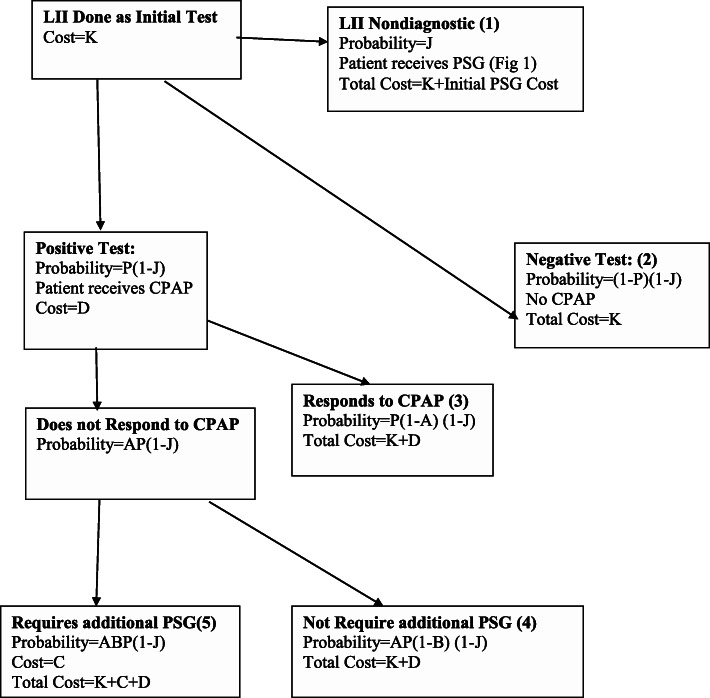
Initial LII Algorithm. Flow of patients through health states if LII was used as the initial test

### Initial LIII algorithm: (Fig. 3)

In this algorithm, LIII was the initial test followed by PSG as required. We made the following additional assumptions:

m) There was a technical failure rate of proportion (E) with a portable study.
n) Ambulatory studies had a sensitivity of F and specificity of G to detect OSA.
o) Patients with a positive ambulatory study were started on CPAP.
p) Patients without OSA did not respond to CPAP (false positives).
q) Patients with a positive ambulatory test but who did not respond to CPAP (either because they didn’t have OSA or because they had OSA and did not respond) obtained a PSG.
r) In patients with OSA who did not tolerate or improve with CPAP, a proportion (H) required only one PSG (i.e. diagnostic) and (1-H) required two PSG (i.e. diagnostic and CPAP titration).
s) Patients with a negative ambulatory study but a positive PSG were considered to have OSA and started on CPAP (false negative).
t) The cost of the LIII study was $I.

**Fig. 3:**
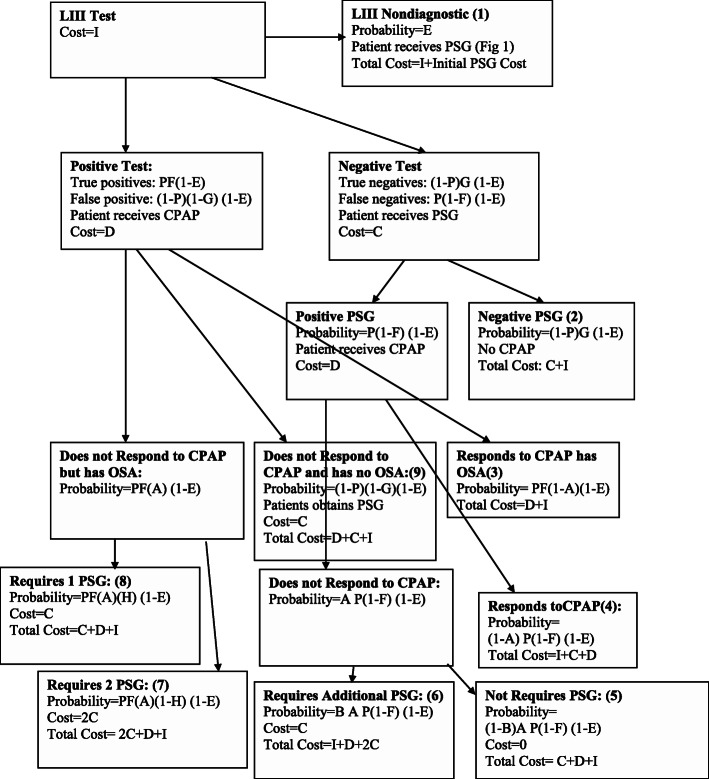
Initial LIII Algorithm (PSG if needed). Flow of patients through health states if LIII was used as the initial test, followed by PSG as required (e.g. to exclude false negatives)

### Initial LIII followed by LII (Fig. 4)

Patients had LIII study initially. If the study was negative OR if the study was positive and the patient did not respond to CPAP, an LII study was done. If there was failure of the LII, then PSG was done.

**Fig. 4:**
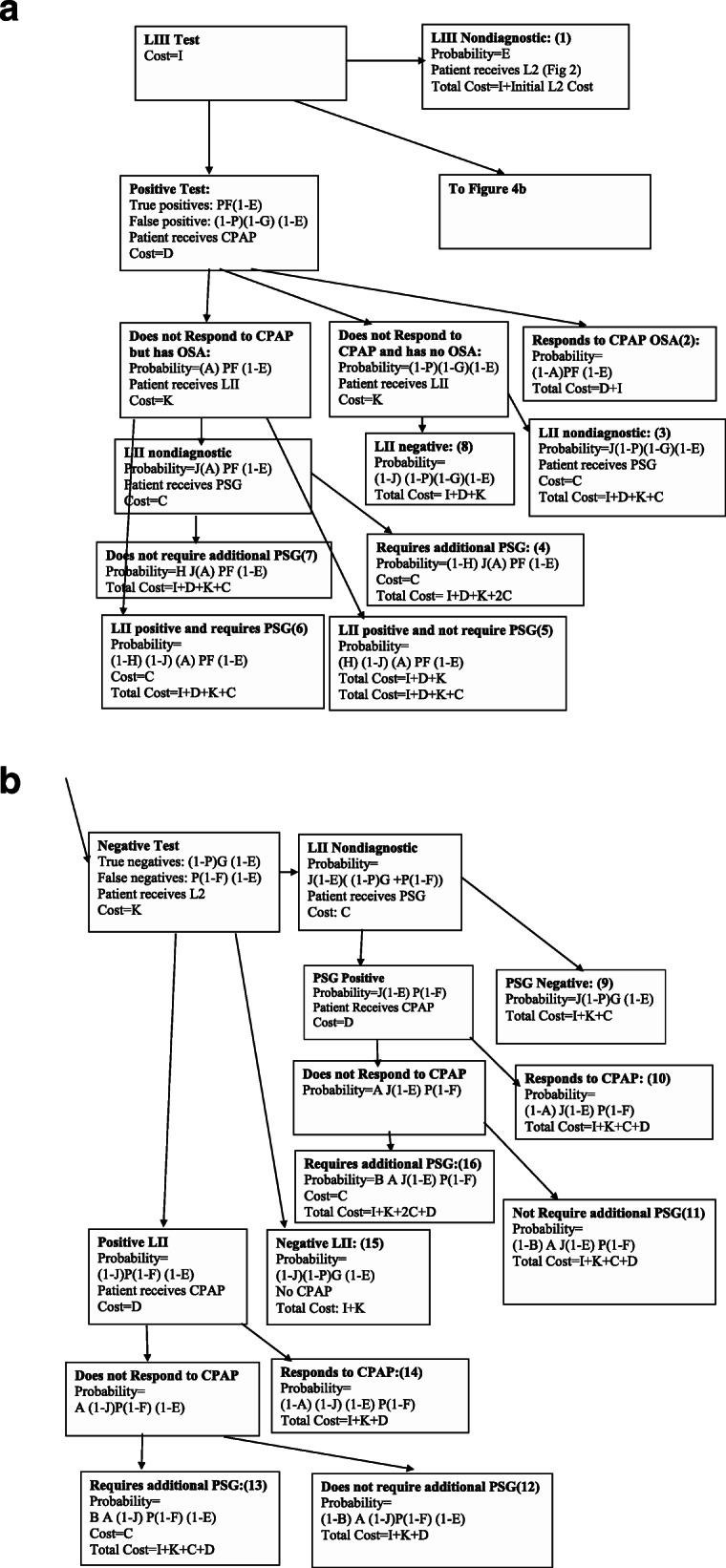
Initial LIII Algorithm (LII if needed). Flow of patients through health states if LIII was used as the initial test, followed by LII as required, and then PSG if necessary

### Illustrative example

To assess how the model might be applied to a specific scenario we considered the situation in British Columbia, Canada. We estimated values for the variables used in our model (Table 1). For some of these values, such as adherence with CPAP (A) (Mehrtash et al. 2019) and sensitivity and specificity of portable devices (Ross et al. 2000), we used data from available literature. For other variables (e.g. proportion of patients intolerant/do not improve on CPAP who require one PSG after their original portable study) we approximated values based upon our clinical expertise.

**Table 1:** Description of Variables Used in the Model and Estimated Values. Costs are in Canadian dollars

Variable	Description of Variable	Estimated Value
A	Proportion of patients with OSA intolerant/do not improve on CPAP	0.3
B	Proportion of patients intolerant/do not improve on CPAP who require PSG after their original PSG/LII	0.4
C	Cost of PSG	$555
D	Cost of CPAP trial	$0
E	Rate of nondiagnostic LIII	0.1
F	Sensitivity of LIII	0.85
G	Specificity of LIII	0.85
H	Proportion of patients intolerant/do not improve on CPAP who require only one PSG/LII after initial LIII	0.6
1-H	Proportion of patients intolerant/do not improve on CPAP who require an additional PSG (after initial portable study and follow up PSG/LII)	0.4
I	Cost of LIII	$167
J	Rate of nondiagnostic LII	0.15
K	Cost of LII	$300

For costs of LI and LIII studies, we used Medical Service Plan 2020 reimbursement rates from British Columbia (sum of both technical and professional fees) (https://www2.gov.bc.ca/assets/gov/health/practitioner-pro/medical-services-plan/msc-payment-schedule-may-2020.pdf 2020). For Level 2 studies, there is no fee code; therefore, we chose a value approximately midway between PSG and Level III studies. In British Columbia, home care companies do not usually charge for a CPAP trial, though device costs are relatively high.

We varied pretest disease probability from 0 (no chance of OSA) to 1 (100% chance of OSA) to determine how this would affect costs of the various algorithms. Calculation of pretest probability involves consideration of the baseline prevalence in the referred population, clinical judgement after history/physical examination, and use of standardized instruments (e.g. STOPBANG, Sleep Apnea Clinical Score) (Chung et al. 2016; Mulgrew et al. 2007b).

## Results

Costs and probabilities according to the various algorithms and variables (Table 1) are shown in Figs. 1, 2, 3 and 4. Cost per patient for each algorithm was calculated by adding the sum of the costs in each of the terminal states multiplied by the probability of arriving in that state. For example, for the PSG algorithm (Fig. 1), cost per patient was the sum of costs and probabilities of boxes 1–4: C (1-P) + (C + D)P (1-A) + (C + D)AP (1-B) + C (2C + D).

### Illustrative example

**Fig. 5:**
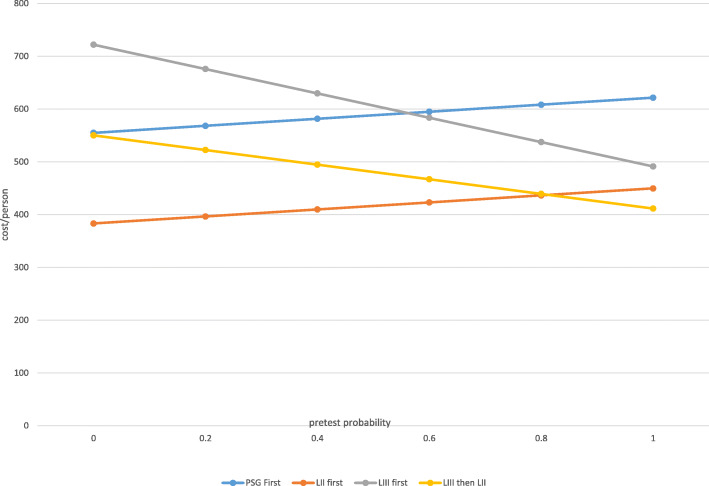
Costs/person by Algorithm and Pretest Probability. Graph showing changes in cost as a function of pretest probability of OSA (from 0- OSA not present; to 1 OSA certain to be present). LIII as initial test more cost efficient at higher pretest OSA probabilities

Using the values in Table 1 for British Columbia, one can see how costs per patient varied according to the four algorithms and pretest probability of OSA (Fig. 5). When initial PSG was compared to initial LIII followed by PSG, PSG was less costly at lower pretest disease probabilities (< 0.6) but LIII was more cost-effective at higher probabilities. This is because a lower pretest probability resulted in more negative LIII tests that then required PSG, thereby increasing costs if LIII was the initial test (Ayas et al. 2010).

The results for LII tests were particularly illuminating. Specifically, Fig. 5 shows that initial LII was less costly than all other algorithms unless pretest probability was very high (i.e., > 0.8). With very high pretest probability, LIII followed by LII was the least costly, though overall magnitude of difference was small compared to LII first ($425 vs. $443 for *P* = 0.9; $408 vs. $412 for *P* = 1.0).

If we consider patients with a pretest probability of 50%, costs per patient for initial PSG, initial LIII followed by PSG, initial LIII followed by LII, and initial LII are as follows: $588, $607, $481, and $417. Therefore, the use of the LII initial algorithm (Fig. 2) could theoretically save approximately $171,000 for every 1000 patients evaluated compared to the initial PSG algorithm (Fig. 1) assuming a LII cost of $300. As a sensitivity analysis, we varied costs of LII studies to assess impacts on algorithm costs assuming a pretest probability of 50%; the LII cost at which the initial PSG and initial LII algorithm were equivalent was $470.

## Discussion

In our theoretical framework, we found that LII studies could be useful in the diagnostic assessment of patients with suspected OSA. The potential utility would vary substantially by a number of factors including: costs of studies (PSG, LIII and LII studies), pretest probability of OSA, CPAP trial costs, and CPAP adherence. An excel spreadsheet allowing calculation of per patient costs according to the algorithm and incorporating these variables can be found in the supplemental materials. In our illustrative case examining the situation in British Columbia, LII were highly cost advantageous across a broad range of pretest OSA probabilities. Incorporating LII may result in substantial cost-savings relative to an initial PSG algorithm. In a sense, this result is not surprising if the data from a LII are equivalent to PSG with lower cost per test.

We acknowledge that there are many limitations to our theoretical model. First, we have described a theoretical model rather than the results of an empiric experiment. Second, we have made a number of assumptions. Although we believe that the assumptions were reasonable, we recognize that some could be challenged (e.g. that LII and PSG were equally effective in diagnosing OSA excluding technical failures, or that all patients with a negative LIII need follow up LII or PSG). Second, certain populations would likely not be appropriate candidates for particular algorithms. For example, patients with substantial underlying heart failure or lung disease should probably not have LIII studies as an initial diagnostic test given the potential presence of non-OSA causes of nocturnal desaturation. Third, our models focused on symptomatic patients in whom tests are done predominately to rule in disease. The role of LIII or LII studies in ruling out disease in low probability patients, assessing patients with mild OSA, and those with substantial cardiopulmonary disease is unclear. Fourth, there is little data about use of LII studies in clinical care. For example, technical failure rates of LII across a broad range of patients is currently unknown. Anecdotally, LII studies are more difficult to set up at home compared to LIII studies. This may be particularly relevant in frail patients, patients with significant arthritic, neurologic or neuromuscular diseases, cognitively impaired patients, or patients who are not particularly technologically inclined. Therefore, these types of patients may not be appropriate candidates for an initial LII study. Furthermore, LII studies generally do not have monitoring of carbon dioxide or video. For certain patients such as suspected seizures or neuromuscular disease with hypoventilation, these data may be required, and LI study would be most appropriate. More empiric data concerning use of LII in clinical situations is required. Fifth, we have not considered costs for physician follow up appointments, costs not associated with medical care (e.g. travel to sleep laboratories, time off work), or preferences (e.g., to have test at home or in the laboratory). Sixth, we have not considered added benefits of LII and PSG over LIII in terms of potential diagnosis of other respiratory (e.g., central sleep apnea) or non-respiratory disorders (e.g., periodic limb movements). Furthermore, LII and PSG could allow assessment of more advanced physiologic metrics that cannot be readily obtained from LIII studies (Younes et al. 2020; Sands et al. 2018). Although these metrics are currently not used in clinical practice, we believe that their use may become more widespread in the future, and help direct more precise precision care (Edwards et al. 2019; Malhotra and Ayas 2020).

## Conclusion

Using a theoretical decision model, we have developed a preliminary framework to assess the potential role of LII studies in the assessment of patients with suspected OSA. Across a broad range of patient pretest probabilities, initial LII studies appear to provide substantial cost advantages. LII studies might be especially useful during pandemics as these tests combine the ability to have the extensive physiologic information characteristic of PSG with the ability to avoid overnight in-laboratory stays.

## Supplementary Information

**Additional file 1.**

## Data Availability

All data generated or analysed during this study are included in this published article.

## Abbreviations

OSA: Obstructive sleep apnea
PSG: Polysomnography
LII: Level II sleep studies
LIII: Level III limited channel home sleep study
CPAP: Continuous positive airway pressure
